# Cortical Double-Opponent Cells in Color Perception: Perceptual Scaling and Chromatic Visual Evoked Potentials

**DOI:** 10.1177/2041669517752715

**Published:** 2018-01-18

**Authors:** Valerie Nunez, Robert M. Shapley, James Gordon

**Affiliations:** Center for Neural Science, New York University, New York, NY, USA; Department of Psychology, 5924 Hunter College, CUNY, New York, NY, USA; Center for Neural Science, New York University, New York, NY, USA; Department of Psychology, 5924 Hunter College, CUNY, New York, NY, USA; Center for Neural Science, New York University, New York, NY, USA

**Keywords:** color perception, saturation, chromatic visual evoked potential, V1, cone contrast

## Abstract

In the early visual cortex V1, there are currently only two known neural substrates for color perception: single-opponent and double-opponent cells. Our aim was to explore the relative contributions of these neurons to color perception. We measured the perceptual scaling of color saturation for equiluminant color checkerboard patterns (designed to stimulate double-opponent neurons preferentially) and uniformly colored squares (designed to stimulate only single-opponent neurons) at several cone contrasts. The spatially integrative responses of single-opponent neurons would produce the same response magnitude for checkerboards as for uniform squares of the same space-averaged cone contrast. However, perceived saturation of color checkerboards was higher than for the corresponding squares. The perceptual results therefore imply that double-opponent cells are involved in color perception of patterns. We also measured the chromatic visual evoked potential (cVEP) produced by the same stimuli; checkerboard cVEPs were much larger than those for corresponding squares, implying that double-opponent cells also contribute to the cVEP response. The total Fourier power of the cVEP grew sublinearly with cone contrast. However, the 6-Hz Fourier component’s power grew linearly with contrast-like saturation perception. This may also indicate that cortical coding of color depends on response dynamics.

## Introduction

The primary visual cortex V1 is a bottleneck for color perception in the cortex; color processes occurring later in the cortex are based on the responses from the neural substrates for color perception in V1. Previous research established that color-responsive neurons in the primary visual cortex, V1, of macaque monkeys can be assigned to color-preferring and color-luminance cell classes (Johnson, Hawken, & Shapley, 2001). Later work showed that color-preferring cells were mostly single-opponent cells and color-luminance cells were mostly double-opponent cells (for a review, see Shapley & Hawken, 2011). As a consequence of this formative work, we now know that all color-responsive neurons in V1 are divided into just two known groups that mediate color: specifically, single- and double-opponent neurons. Therefore, if a V1 response to color is not due to single-opponent neurons, it must be due to double-opponent neurons, and vice versa.

Given that in V1, the only color-responsive neurons are single- and double-opponent cells, we sought to answer the fundamental question, what is the relative contribution of each class of cells to color perception (cf. different opinions published previously: Shapley, Hawken, & Johnson, 2014; Solomon & Lennie, 2007)?

Single- and double-opponent V1 neurons have quite different selectivities for spatial patterns of color. Single-opponent cells respond best to equiluminant color patterns with spatial frequency <0.5 c/deg and not at all to color patterns >2 c/deg, while double-opponent cells respond best to color patterns at 2 c/deg and very little <0.5 c/deg (Johnson et al., 2001; Lennie, Krauskopf, & Sclar, 1990; Schluppeck & Engel, 2002; Shapley et al., 2014; Thorell, De Valois, & Albrecht, 1984). The different spatial properties allow experimenters to stimulate only one class of color-responsive cells by choosing a pattern that is not visible to the other class.

To answer the question, to what extent do different cell classes contribute to color perception, we measured the color appearance of fine checkerboard patterns (comprising equiluminant color checks) that have fundamental spatial frequencies around 2 c/deg. Such patterns should activate V1 double-opponent cells preferentially. We also measured color appearance of large, uniform color squares that should activate the single-opponent cells in V1. The participants’ color experience was estimated with saturation scaling (see Methods section). The perceived color saturation of both the checkerboards and the uniform squares increased with increasing cone contrast. However, participants assigned larger saturation values in response to an equiluminant color-gray checkerboard than to a uniform square of the equivalent size and cone contrast (see Results section). These results suggest that for stimuli designed to stimulate double-opponent cells optimally, the amount of color observed was greater than the amount of color in stimuli chosen to excite only single-opponent cells. Previously, it was hypothesized that double-opponent cells were important for color contrast (Livingstone & Hubel, 1984) or color constancy (Gegenfurtner, 2003). Now, we are compelled by the data to propose that double-opponent cells have a major role in the perception of color in most spatial patterns. More about our proposal is in the Discussion section.

A second aim was to compare behavioral and electrophysiological measures of color responsiveness in humans. We compared behavioral, perceptual-scaling data with the responses to color of neuronal populations in human V1 cortex by measuring the chromatic visual evoked potential, the cVEP (Crognale, 2002; Crognale, Duncan, Shoenhard, Peterson, & Berryhill, 2013; Murray, Parry, Carden, & Kulikowski, 1987; Rabin, Switkes, Crognale, Schneck, & Adams, 1994; Souza et al., 2008), over the same range of color contrast and for the same stimulus patterns as in the behavioral experiments. As reported in the Results section, the cVEP response to equiluminant checkerboard patterns was much bigger than the response to large, uniform, equiluminant color squares, in agreement with much of the earlier work on the spatial selectivity of the cVEP (Murray et al., 1987; Porciatti & Sartucci, 1996; Rabin et al., 1994). Our results and the earlier results on cVEP spatial selectivity, combined with the known spatial properties of V1 single- and double-opponent cells (e.g., Schluppeck & Engel, 2002), imply that the checkerboard cVEP is driven by V1 double-opponent cells. Comparison of the cVEP and color saturation perception was not straightforward and led to interesting results.

## Methods

### Participants

All observers gave written informed consent to participate in this study. The experiments were conducted in accordance with the principles embodied in the Declaration of Helsinki and were approved by the Hunter College/City University of New York and the New York University institutional review boards.

Nine observers (three male, six female) aged 19 to 48 years (*M* = 27, *SD* = 10) participated in this experiment. All participants had normal color vision, assessed with the 18-plate series Pseudo-isochromatic Plates for Testing Color Perception compiled in 1940 by the American Optical Company; Farnsworth dichotomous test for color blindness—Panel D15; Lanthony’s desaturated 15 hue test; and the Farnsworth-Munsell 100-hue test for color vision. The participants also had at least 20/20 (or corrected to 20/20) visual acuity, measured using a Snellen chart at 114 cm (the distance to the screen during experiments).

### Visual Stimuli

A Sony PVM-1741A OLED monitor was used to present the stimuli. The monitor had a diagonal screen size of 42 cm, resolution of 1920 × 1080, and vertical refresh rate of 60 Hz. The screen was calibrated using a Photo Research PR670 Spectrascan Radiometer/Photometer, and this was used to calculate a gamma correction to linearize the screen output to ensure complete control of the intensities on the screen.

**Figure 1.**
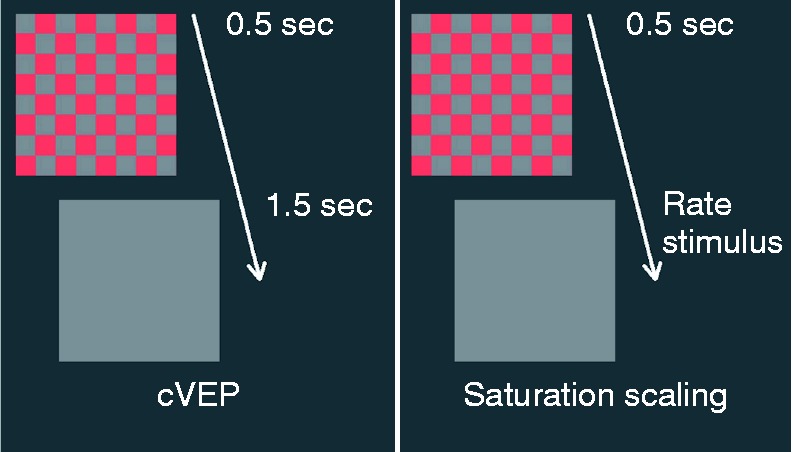
Representation of the appearance–disappearance stimulus with time during the cVEP and scaling experiments. For 0.5 s, a checkerboard of squares was visible. In the checkerboard, squares of equiluminant red of a specific cone contrast alternated with squares that were gray like the background. This “on” pattern consisted of 32 × 32 squares covering 10° × 10° of arc subtended at the eye and was followed by an interval during which the entire screen was uniform gray (“off”). The transition between “on” and “off” patterns occurred suddenly; thus, the temporal modulation signal was a rectangular wave. All checks and the background were the same luminance. During cVEP experiments, the “off” pattern of duration was 1.5 s, with the “on/off” cycle being repeated 30 times per stimulus. During scaling experiments, the “off” pattern lasted until the participant had completed the saturation ratings for the stimulus, after which the next stimulus was presented.

The stimuli for both the perception (scaling) experiments and for measuring cVEPs were equiluminant red–gray color checkerboard patterns and large, uniformly colored red squares embedded in an equiluminant gray background. The stimulus size was 20 cm × 20 cm which at a distance of 114 cm corresponded to 10° × 10° of arc subtended at the eye. For the perceptual scaling experiments, each pattern appeared for 0.5 s and then disappeared, at which time the participant rated the color saturation. In the cVEP experiments, the pattern appeared for 0.5 s and then disappeared for 1.5 s, with this cycle being repeated in a block of 30 trials (lasting a total of 60 s) for each stimulus. This rectangular-wave modulation from gray background to color and back to gray (0.5 s on, 1.5 s off; i.e., modulated at 0.5 Hz with a duty cycle 0.25) is so-called appearance-disappearance modulation. The temporal modulations of the stimuli for both experiments are represented in Figure 1. The checkerboard had 32 × 32 checks. Therefore, each check spanned 0.3125° of arc, for which the dominant spatial frequency has a period of 0.3125 × √2 = 0.4419°, giving a dominant spatial frequency of 1/0.4419 = 2.26 cycles per degree, near the peak of the spatial frequency response reported by Rabin et al. (1994). The background gray color corresponded to a color temperature of 5800 K and the pattern color was one of six saturation levels of red along the direction in CIE space from the white point to the red phosphor of the screen, with root mean square (RMS) cone contrast ranging from 0.03 to 0.40. The spatio-chromatic stimuli were chosen so that participants could perceive a definite color in the colored checks and squares for the hue and saturation scaling experiments (Gordon, Abramov, & Chan, 1994). The corresponding chromatic excitation purities and CIE coordinates are provided in Table 1. For all stimuli, the luminance was 31 cd/m^2^.

**Table 1.** RMS Cone Contrast and Corresponding Color Excitation Purity and CIE Color Coordinates for Each Stimulus Presented.

		CIE color coordinates	
Cone contrast	Color excitation purity	x	y
0.028	0.051	0.334	0.335
0.086	0.133	0.363	0.335
0.13	0.196	0.384	0.336
0.18	0.264	0.408	0.336
0.27	0.399	0.454	0.337
0.40	0.593	0.522	0.336

Stimulus presentation was controlled using the Psychophysics Toolbox extensions (Brainard, 1997; Kleiner et al., 2007; Pelli, 1997) for Matlab R2012b (The MathWorks, Inc., Natick, MA, USA), which ran on a Dell Inspiron-3847 computer using the Microsoft Windows 7 operating system. To ensure tighter control of timing, particularly for changing images on the screen from one frame to the next, we followed methods similar to those proposed by Scarfe (n.d.). A trigger signal was sent via the serial port to the recording system directly before each stimulus was presented.

During each experiment, the participant was seated such that her or his eye level was aligned with the center of the screen and the viewing distance was 114 cm. Stimuli were viewed binocularly. During cVEP runs there was one block of stimulus presentations for each cone contrast and the blocks were presented in random order. Each participant was asked to focus on the center of the screen and to blink as little as possible, particularly when a stimulus was visible on the screen.

### Saturation Scaling

We employed a form of hue and saturation scaling used by Gordon et al. (1994) derived from Jameson and Hurvich (1959). The technique is essentially the same that we have used before in other experiments (Gordon et al., 1994; Gordon & Shapley, 2006; Xing, Ouni, Chen, Sahmoud, Gordon, & Shapley, 2015). For the purposes of this report, we consider only saturation ratings. Each stimulus was presented a total of four times in random order for rating purposes. For each stimulus presentation, the screen showed the gray background, and then the stimulus was presented for a duration of 0.5 s before the screen was switched back to the gray background (as in Figure 1). The observers described saturation as the percentage of the entire sensation, chromatic and achromatic, that was chromatic. The experimenter explained that the total absence of hue (i.e., gray) would be represented by 0% saturation, and a total absence of any achromatic sensation (i.e., pure color) would be 100% saturation. Participants were given as many practice trials beforehand as were needed for them to be comfortable with the scaling process, usually corresponding to a minimum of 10 practice trials.

Saturation can be a problematic concept for participants. In spite of the difficulty, we have obtained consistent saturation scaling across observers (for instance in Gordon & Shapley, 2006). Many other groups have studied color appearance using similar hue and/or saturation scaling methods (e.g., Bimler, Paramei, & Izmailov, 2009; De Valois, De Valois, Switkes, & Mahon, 1997; Knau & Werner, 2002; Schultz, Doerschner, & Maloney, 2006; Volbrecht & Nerger, 2012).

### cVEP Data Acquisition

Data were recorded using a BioSemi ActiveTwo system (BioSemi, Amsterdam, Netherlands); with 64 electrodes, we obtained the spatial resolution of a 128-channel system by positioning 63 electrodes on the back half of a 128-channel BioSemi electrode cap setup with the extended 10-20 system (based on the Oostenveld and Praamstra (2001) 5% system). One electrode was positioned at Fpz and all data were re-referenced to Fpz after data acquisition. When aligning the electrode cap, we ensured that the electrode for Oz was correctly positioned at 10% of the inion-nasion distance along the midline of the scalp. The trigger and electroencephalography (EEG) signals were sampled at a frequency of 2048 Hz, with an open passband from 0 to 400 Hz. Usually, each experimental run consisted of 30 trials = periods of the 0.5 Hz stimulus. Thus, each cVEP point is usually based on 60 s worth of data.

### cVEP Data Analysis

Using functions from the FieldTrip toolbox for EEG/MEG analysis (Oostenveld, Fries, Maris, & Schoffelen, 2011; http://www.ru.nl/neuroimaging/fieldtrip), we imported the response data for each stimulus and separated them into trials containing a prestimulus period of 100 ms and poststimulus period of 500 ms.

The EEG data in each trial were re-referenced with respect to electrode Fpz, and then were baseline-corrected with respect to the average voltage across each entire trial. The data were inspected visually (all channels simultaneously on a trial-by-trial basis) to remove blinks and artifacts due to movement or extreme electronic noise transients (greater than 150 µV). At this point, the trial data were baseline-corrected with respect to the prestimulus period and were grouped into epochs of three trials before a Discrete Fourier Transform was calculated, using a period of 0.5 s, covering the duration of the stimulus. This resulted in a Fourier fundamental frequency of 2 Hz. Note that we chose not to conduct Fourier analysis over the whole stimulus on–off period because our purpose was strictly to focus on the waveform shape over the time when the stimulus was visible. This was partly because the participants tended to blink more after the stimulus disappeared but mainly because the responses in which we are interested were in this range. The first 100 Fourier harmonics were used to construct inverse FT waveforms. Before the reconstruction step, the data were filtered for 60 Hz noise and its harmonics by setting the amplitudes of the corresponding harmonics to zero. No additional filtering, including any high-pass band filtering, took place. Note that when further analysis involved the grand averaging of Fourier data across all participants, a few data points were excluded from the averaging due to excessive electrode noise or drift for a given participant or stimulus.

## Results

### Perceptual Scaling Results

**Figure 2.**
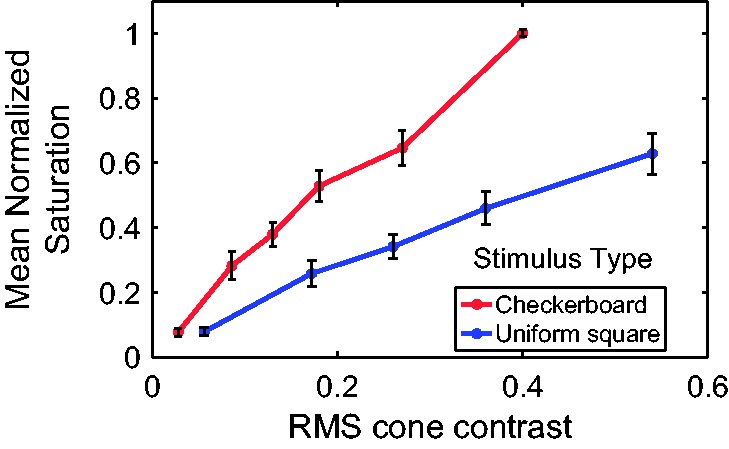
Normalized saturation scaling data averaged across all participants, presented as a function of RMS cone contrast for the checkerboard (red) and uniform colored square (blue) patterns. Error bars represent ±1 *SEM*. Note that the cone contrast of the uniform square was scaled to take into account the fact that the space-averaged cone contrast of the red–gray checkerboard was one-half that of a uniform square of the same chroma or cone contrast (compared with the equiluminant gray).

Normalized saturation scaling data averaged across all participants are drawn in Figure 2. The color of the red checks in the high-frequency checkerboard scaled in saturation fairly linearly with cone contrast. Participants’ scaling data were very similar to one another as indicated by the small error bars in Figure 2. For the range of cone contrasts used, there is no evidence of the ratings hitting a response ceiling with increasing cone contrast; the perceived saturation grew monotonically and directly proportional to the cone contrast.

Perceived saturation of color checkerboards was higher than that of a large, uniform square of the same space-averaged cone contrast (Figure 2). Why did we compare the checkerboard appearance to that of a square of equivalent space-averaged contrast? The reason is, if the responses to both types of stimuli were due only to contributions from single-opponent neurons, the response to the fine checkerboard would be to its space-averaged cone contrast because single-opponent cells cannot resolve such fine patterns (see Introduction section). Therefore, to test the hypothesis that all color perception must go through the single-opponent cells, one ought to compute the spatial average of the checkerboard cone contrast to match the responses from the square. The fact that the checkerboard pattern evokes a greater response than the square of equivalent cone contrast suggests that there must be a visual response to color in addition to that of single-opponent neurons. In the early visual cortex V1, the only other possible neural substrate of color perception is the population of double-opponent neurons. The space-averaged cone contrast of a red-gray checkerboard is one-half that of a large, uniform square of the same chroma or cone contrast (compared with the equiluminant gray). Therefore, for the checkerboard, we plotted the data at the same *x*-axis value as the square that had half the cone contrast. Figure 2 reveals that color checkerboards were especially good stimuli for evoking the appearance of color and that much of their color appearance must be caused by higher frequency components in the color checkerboard rather than the space-averaged DC component. Since the checkerboard pattern is fairly fine and would be sensed much better by double-opponent than by single-opponent cells in V1 cortex (Schluppeck & Engel, 2002), Figure 2 is evidence that double-opponent cells contribute very significantly to color perception.

### cVEPs: Waveforms and Topography

The cVEP waveforms were predominantly negative deflections, consistent with earlier reports (Crognale, 2002; Crognale et al., 2013; Murray et al., 1987; Rabin et al., 1994; Souza et al., 2008). The cVEP was recorded with a dense multielectrode array (Methods section) from which we could estimate the regions of the cerebral cortex activated by the spatio-chromatic stimulus. The waveforms and topography of the cVEP responses to the checkerboard were discussed in detail in previous work (Nunez, Shapley, & Gordon, 2017). The conclusion from our analysis of cVEP topography was that most of the cortical activity we studied was generated within primary visual cortex, V1. This was in agreement with work of other researchers (e.g., Crognale et al., 2013; Xing et al., 2015). There was no notable activity in extra-striate cortex or in nonvisual cortex preceding the rise of the cVEP to its peak. Finally, the largest cortical response was consistently observed at Oz, also consistent with prior work (e.g., Murray et al., 1987). As a result, data in this article focus solely on cVEP recordings from electrode Oz.

### cVEPs: Nonlinear Dynamics With Color Contrast

**Figure 3.**
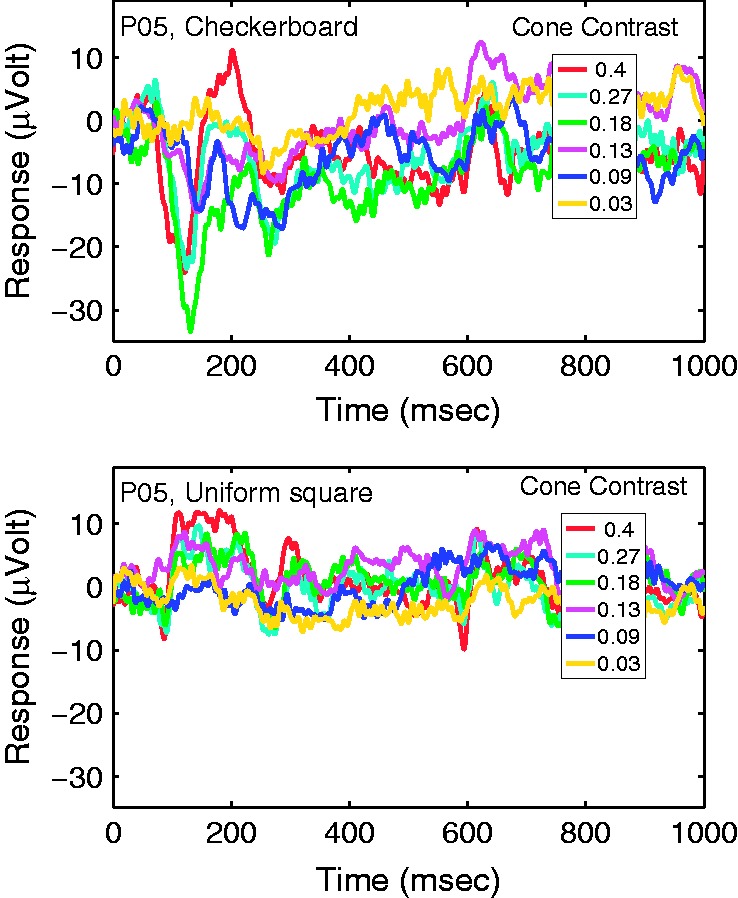
The cVEP waveform at electrode Oz for a typical participant observing the checkerboard (top) and uniform-square (bottom) patterns. For both stimuli, responses are plotted for a selection of cone contrasts, covering the time period from pattern onset to 1000 ms after pattern onset. Note that the pattern was visible only from 0 to 500 ms.

Figure 3 depicts the cVEPs of one participant over a range of cone contrasts to illustrate cVEP waveforms and dependence on cone contrast. In the upper panel are responses to checkerboard patterns; in the lower panel are responses to large, uniform squares, also over a range of cone contrasts. The results (Figure 3) support earlier findings that the cVEP is spatially tuned; the cVEP is much larger in responses to equiluminant patterns of intermediate spatial frequency and its response is much weaker to low frequency or spatially uniform color stimuli (Murray et al., 1987; Porciatti & Sartucci, 1996; Rabin et al., 1994; Tobimatsu, Tomoda, & Kato, 1995).

The cVEP generated in V1 by the checkerboard patterns exhibited nonlinear dynamics in responses to different cone contrasts, that is, the temporal waveform of the response changed dramatically from low to high cone contrast (Nunez et al., 2017). The cVEP for lower cone contrast was (a) slower to rise (Crognale et al., 1993; Porciatti & Sartucci, 1996; Rabin et al., 1994; Souza et al., 2008) and (b) more prolonged than at higher contrast. We analyzed these nonlinear dynamics further in a number of ways. We replicated results on shorter cVEP latency at higher cone contrast (Crognale et al., 1993; Porciatti & Sartucci, 1996; Rabin et al., 1994; Souza et al., 2008) by Fourier analyzing the cVEP waveform into harmonics of a fundamental frequency, 2 Hz, which is the frequency that has 0.5 s, the duration of the stimulus, as its period (see Methods section). The largest harmonic amplitude was usually at 4 Hz, so we analyzed the phase shift of the 4 Hz component in the Fourier transform of the cVEP. Consistently across all observers, there was a very large phase advance, greater than or equal to 100° of phase, from low to high cone contrast (Nunez et al., 2017), replicating results of many previous studies that found decreasing latency with increasing cone contrast (Crognale et al., 1993; Porciatti & Sartucci, 1996; Rabin et al., 1994; Souza et al., 2008).

We analyzed the Fourier representation of the checkerboard responses further (see Methods section). The main analysis was of the contrast dependence of the amplitude spectrum of the cVEP. This analysis extended only up to the first five harmonics of the fundamental frequency based on an analysis of the cumulative power spectrum (e.g., Jospin et al., 2007), the sum of the power up to and including a specified harmonic. Cumulative power spectra indicated that most of the power of the cVEP was contained in Harmonics 1 to 5 under all conditions (Nunez et al., 2017).

**Figure 4.**
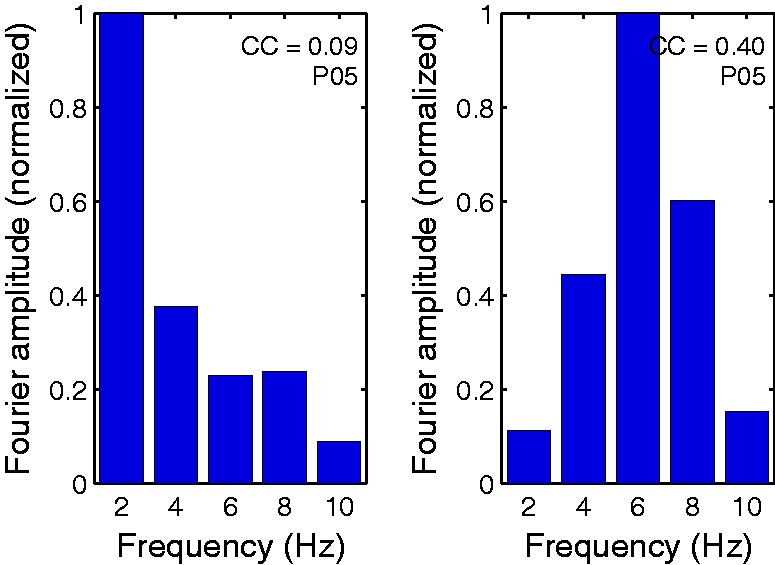
Normalized Fourier amplitude spectrum of checkerboard responses for two different RMS cone contrasts (0.09 on the left and 0.4 on the right) for a typical participant.

Analysis of Fourier amplitude spectra of the color-checkerboard-evoked cVEP waveforms demonstrates the profound change in response dynamics with cone contrast. Fourier amplitude spectra are drawn in Figure 4 for one representative participant’s data. The Fourier spectra were normalized to 1 at peak amplitude. Figure 4 shows the spectra change with cone contrast; there is much more power in the higher harmonics at high contrast than at low. This change of spectrum with contrast is a nonlinear effect (cf. Nunez et al., 2017).

### Color Perception and cVEP Comparison

**Figure 5.**
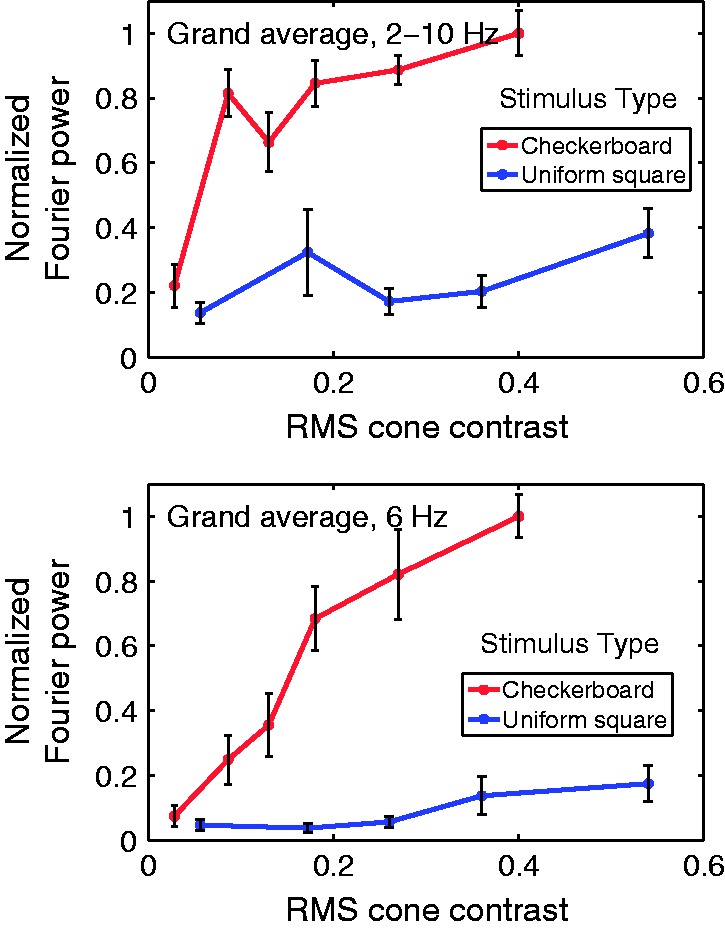
Normalized Fourier power averaged over all participants and plotted as a function of RMS cone contrast for the checkerboard (red) and uniform-square (blue) stimuli. In the top graph, the Fourier power for each participant was summed over the first five Fourier harmonics (2 to 10 Hz) before normalization and grand averaging. The lower graph shows normalized Fourier power calculated for only the third harmonic (corresponding to 6 Hz) before normalization and grand averaging. Note that the error bars represent ±1 *SEM*.

Fourier analysis also was used to study the dependence of cVEP response power on cone contrast (Figure 5). Figure 5 top panel depicts response power versus cone contrast averaged across all nine participants in this study: power summed across the first five harmonics (2–10 Hz) of the stimulus period. As we reasoned earlier, the cumulative power spectra indicated that most response power was contained in these five harmonics so the power summed over them should give a good estimate of total response power (Parseval’s Theorem). Two separate contrast-response functions are graphed for the two kinds of stimuli used: (a) checkerboard and (b) large, uniformly colored square. For the checkerboard responses, summed power (2–10 Hz) rises steeply between 0.03 and 0.09 cone contrast and then levels off so that response power at 0.09 contrast is already 80% as large as the response to the highest cone contrast used, 0.4. In other words, response power of the response to color checkerboards grows sublinear with cone contrast. Also in Figure 5 upper panel, the data for total power of the response to large, uniformly colored squares are plotted. Consistent with the spatial tuning evident in our data (Figure 3), the total response power of the responses to the large, uniform red squares was much less than that of the checkerboard responses at the same space-averaged cone contrast.

The cVEP power summed across the first five harmonics (that we had previously ascertained as constituting the response power of the cVEP, as stated before) did not track the perception (scaling data in Figure 2) but rather rose more quickly with cone contrast than the scaling data, and flattened at cone contrast > 0.1. This flattening of cVEP power with cone contrast replicates earlier results on sublinear response versus color contrast. Peak amplitude of the cVEP versus contrast was previously found to be roughly proportional to the log of the cone contrast (Fiorentini, Burr, & Morrone, 1991; Gomes et al., 2010; Souza et al., 2008; Xing et al., 2015).

When we plotted individual Fourier component power versus cone contrast for the checkerboard data (for a range of individual harmonics as well as for various harmonic combinations), we found that the dependence of the power of the 6 Hz (H3) component was roughly proportional to cone contrast like the saturation scaling data (Figure 2). This result is accentuated in Table 2 which presents the correlation coefficients for Fourier power versus saturation rating for each participant, where Fourier power was calculated for just the 6 Hz component as well as for the combined 2 to 10 Hz Fourier components. For seven of the nine participants, the correlation coefficients were higher for the 6 Hz component than for the total 2 to 10 Hz Fourier components.

**Table 2.** Correlation Coefficient Calculated Between Total Power of Given Fourier Component(s) and Saturation Rating When Viewing Checkerboard Stimuli, for Each Participant, P.

Fourier component(s)	Participant								
	P1	P2	P3	P4	P5	P6	P7	P8	P9
6 Hz	0.88	0.88	0.86	0.78	0.89	0.73	0.59	0.85	0.72
2 to 10 Hz	0.76	0.99	0.78	0.50	0.88	0.90	0.03	0.39	0.62

*Note.* Note that the correlations between participants reflect individual differences in cVEP results and saturation ratings across the range of cone contrasts.

It is possible that this result means that a dynamic component of the neuronal population activity is more salient for perception than the total power or peak response, as we will discuss later.

## Discussion

### Scaling and Color Perception—Double-Opponent Versus Single-Opponent Cells as the Source of Color Perception

It is important to consider the neural origins of color appearance. We want to consider here the color-responsive neurons in V1 because color signals must be processed in V1 on the way to color responsive neurons in other cortical areas. The color checkerboards that were our stimuli were equiluminant color checkerboards; therefore, they evoke responses only from subpopulations of V1 neurons that are responsive to color: single-opponent and double-opponent cortical neurons (Schluppeck & Engel, 2002).

It is possible to infer what population of color-responsive neurons supports color appearance based on previous work on the spatial frequency sensitivity and selectivity of neurons in macaque monkey V1 (Johnson et al., 2001; Johnson, Hawken, & Shapley, 2004; Lennie et al., 1990; Schluppeck & Engel, 2002; Thorell et al., 1984; reviewed in Shapley et al., 2014). Cortical color computations are based on the combined activity of two kinds of cortical cone-opponent neurons, single- and double-opponent cells, and also on the cone-nonopponent neurons that respond strongly to achromatic patterns (reviewed in Shapley et al., 2014). Single-opponent cells integrate and double-opponent cells differentiate color signals across visual space. Single-opponent cells respond to large areas of color and to the interiors of large patches. Double-opponent cells respond to color patterns (Johnson et al., 2001) and color boundaries (Friedman, Zhou, & von der Heydt, 2003). Single- and double-opponent neurons have different spatial frequency responses and this fact can be used to test their contributions to color appearance. As shown by Schluppeck and Engel (2002), single-opponent neurons not only respond to lower spatial frequencies but their responses cut off at lower spatial frequencies than those of double-opponent cells. The spatial frequency tuning of double-opponent cells (and also nonopponent cells) are spatially band pass (Schluppeck & Engel, 2002). There is a spatial frequency range (1 to 4 c/deg) where double-opponent cells respond and single-opponent neurons respond weakly or not at all. In this range, color stimuli are mainly stimulating double-opponent neurons. This is the range in which we measured saturation scaling with the checkerboards, by design exploring whether or not color was perceived in moderately fine patterns when one would expect that only double-opponent cells contributed to the percept. Under these conditions, there was reliable color scaling that was roughly proportional to cone contrast in all our participants and that was in fact considerably larger than the perceived saturation of uniformly colored squares of the same space-averaged cone contrast. These results are evidence that double-opponent cells indeed contribute to color appearance over the full gamut of cone contrasts studied.

There was a DC component of color in the red–gray checkerboard patterns that could have been an effective stimulus for single-opponent cells. The DC component is half of the strength of a large, uniformly colored square stimulus of the same chroma as the checks in the checkerboard because half of the checks were neutral gray and the chromatic DC component is the average of the chroma in the red and gray checks. In the scaling experiments with large, uniformly colored square stimuli (Figure 2), we found that the perceived saturation of the DC component was much less than the saturation perceived in the checkerboard so that the major contributor to the perceived color of the checks was a neural mechanism that responded to the checks—and we conclude that this is the double-opponent population (because there are no other color-responsive neurons in V1). These results led to our proposal that double-opponent cells are major contributors to the perception of color in all color patterns. Most double-opponent cells in V1 respond to both color and achromatic luminance patterns (Johnson et al., 2001, 2004). If double-opponent cells comprise the neuronal substrate of color perception in spatial patterns, as we propose, cortical decoding of population activity would be necessary to recover the stimulus color (discussed in Shapley & Hawken, 2011).

### V1 Double-Opponent Cells as the Source of cVEP Signals

To compare the electrophysiologically recorded cVEP signals with color perception, one needs to infer what is the source of the cVEP signal. We found that for spatial patterns such as checkerboard patterns, the cVEP was localized mostly over posterior occipital cortex (Nunez et al., 2017) consistent with the hypothesis that the cVEP is generated in V1 cortex (Crognale et al., 2013; Xing et al., 2015). More evidence that the cVEP reflects color-evoked activity in the primary visual cortex is as follows. The cVEP does not vary with attention, a result that strongly suggests it is evoked early in cortical visual processing (Highsmith & Crognale, 2010). Furthermore, normal cVEPs have been recorded in cases of cerebral achromatopsia where color appearance was lost and lesions were observed in ventromedial extrastriate cortex, but V1 responses to color were unaffected by the lesion (Crognale et al., 2013; Victor, Maiese, Shapley, Sidtis, & Gazzaniga, 1989). The combined evidence from source localization, lack of attentional effects, and cerebral achromatopsia indicates that the cVEP is an index of early cortical responses to color, and the topography (Nunez et al., 2017) supports this conclusion. However, for the low contrast cVEP, there seems to be spreading activity to lateral posterior cortical areas indicating that the cVEP may involve extra-striate cortex at low cone contrast (Nunez et al., 2017).

Double-opponent cells comprise approximately 80% of all color responsive cells in the output layers two thirds of macaque V1 cortex (Friedman et al., 2003; Johnson et al., 2001) and that may be why they might contribute most to the cVEP signal. It also is noteworthy that macaque cortical responses to color measured with voltage-sensitive-dye-imaging or VSDI also are consistent with a preponderance of edge-sensitive double-opponent cells in primate V1 cortex (Zweig, Zurawel, Shapley, & Slovin, 2015).

It is well known that the cVEP signal is tuned for spatial frequency like double-opponent cells and unlike single-opponent cells. cVEP amplitude is much smaller for lower spatial frequencies than it is at its peak spatial frequency, between 1 and 2 c/deg, a consistent result across many studies of cVEP (Murray et al., 1987; Porciatti & Sartucci, 1996; Rabin et al., 1994; Tobimatsu et al., 1995). Our results in Figure 5, about total response power for cVEP responses to checkerboards versus those to large, uniform squares, strongly support the earlier findings about spatial tuning of the cVEP. Taken together with results from single-cell recording in primates (Johnson et al., 2001; Schluppeck & Engel, 2002), the spatial tuning of the cVEP suggests that it is mainly driven by V1 double-opponent cells that also are spatially tuned. Unlike double-opponent cells, cortical single-opponent cells respond best to patterns of low spatial frequency or to uniform fields of color (Johnson et al., 2001; Lennie et al., 1990; Shapley et al., 2014; Thorell et al., 1984).

### Why Do Scaling and 6-Hz cVEP Component Have Similar Contrast-Response Functions

The results on the sublinear cone contrast dependence of total cVEP power confirm results of earlier studies (Fiorentini et al., 1991; Souza et al., 2008; Xing et al., 2015). The simplest possible coding of apparent saturation as the integration of the total power of neuronal responses in V1 is not supported by the earlier studies or by our own results. However, when we dissected the cVEP response into Fourier components, surprisingly we found that the power of the component at 6 Hz was linear with cone contrast like saturation perception. Thus, we can propose the hypothesis that color saturation is encoded in the 6 Hz component of the cVEP. This hypothesis needs to be tested with a wide range of color stimuli in the future. The hypothesis could be a powerful tool for linking neuronal responsiveness to color perception.

Still, it is mysterious why a dynamic component of the response should be what the brain is using for gauging color saturation. The most straightforward explanation is that the cortical readout of V1 activity has a temporal resonance or tuned filter that is especially sensitive to 6 Hz or frequencies near it. This can only be speculation at this point in time, but the curious result about perception and 6 Hz could be a useful clue to mechanisms that link neural activity to perception.
